# Aplastic Anaemia in Pregnancy: A Case-Based Comprehensive Review of the Literature

**DOI:** 10.7759/cureus.58365

**Published:** 2024-04-16

**Authors:** Adela Perolla, Blerina Cela, Valentina Semanaj, Teuta Dedej-Kurti, Tatjana Caja

**Affiliations:** 1 Internal Medicine/Haematology, University of Medicine, Tirana, ALB; 2 Internal Medicine/Haematology, University Hospital Center "Mother Teresa", Tirana, ALB; 3 Pathology and Laboratory Medicine, University Hospital Center "Mother Teresa", Tirana, ALB; 4 Laboratory Medicine, University Hospital Center "Mother Teresa", Tirana, ALB

**Keywords:** aplastic anaemia, maternal-foetal health, haematologic disorders in pregnancy, obstetric complications, bone marrow failure, pregnancy

## Abstract

Aplastic anaemia (AA) is a rare and life-threatening haematologic disorder characterised by pancytopenia and bone marrow failure. Its occurrence during pregnancy is exceedingly rare, posing significant risks and management challenges for both the mother and the foetus. We present here the case of a 23-year-old female, six months pregnant, diagnosed with severe aplastic anaemia (AA), aiming to highlight the diagnostic challenges and management considerations of AA in pregnancy. Our case underscores the critical nature of considering aplastic anaemia in differential diagnosis for pregnant patients presenting with unexplained pancytopenia. Based on that, we performed a comprehensive literature review of the past 20 years of papers published in the English language identified through searches in PubMed, Medical Literature Analysis and Retrieval System Online (MEDLINE), Embase and the Cochrane Library, to provide an in-depth analysis of the current understanding of AA in pregnancy. We emphasise the necessity for cautious yet thorough investigation in such cases to avoid complications in both maternal and foetal health, focusing attention on the need for further research into safe and effective treatment protocols for managing AA in pregnancy, given the complexities introduced by the condition and its treatment on pregnancy outcomes.

## Introduction

Aplastic anaemia (AA) stands as a rare, potentially fatal blood disorder marked by a decrease in all types of blood cells (red cells, white cells and platelets {PLT}) due to bone marrow failure [1]. This condition was first identified by Paul Ehrlich, a Nobel Prize-winning German physician and scientist, in 1885 during a post-mortem examination of a young pregnant female who died after a rapid, severe illness. It was not until 1904 that the term 'aplastic anaemia' was coined by Anatole Chauffard [2]. The quest for understanding AA's diagnosis and treatment has continued since its discovery. In Western nations, AA's incidence is about 2-5 cases per million individuals annually [3-5], but this rate is notably higher in Asia, with a 4-5 times greater incidence [6,7]. In a study performed in Albania, the rate was found lower resulting in 1.35 per million inhabitants [8]. 

The origins of AA during pregnancy are mostly unknown, though potential triggers include exposure to certain chemicals, radiation, drugs, viral infections and factors related to autoimmunity or genetics [1]. The disease's underlying process involves the inability of haematopoietic stem cells to evolve into mature blood cells, leading to pancytopenia and an absence of bone marrow [1]. Whilst the specific reasons behind AA in pregnancy are yet to be fully deciphered, it is suggested that the immune changes associated with pregnancy might contribute to the onset or worsening of the condition [9,10].

Diagnosing and treating AA in pregnant females are particularly challenging due to symptoms that may mimic normal pregnancy alterations, such as anaemia and low platelet counts [9-11]. Additionally, establishing an effective treatment regimen for pregnant AA patients is complicated by the condition's rarity and the potential dangers posed by standard therapies used in nonpregnant individuals [12].

Considering the scarcity and intricacies of AA in the context of pregnancy, a thorough literature review is essential for advancing our comprehension and management strategies for this difficult situation. This analysis seeks to delve into the current literature concerning AA in pregnancy, with an emphasis on its causes, underlying mechanisms, diagnostic approaches, treatments and patient outcomes whilst also highlighting areas in need of further investigation.

## Case presentation

A 23-year-old pregnant female, six months into her term, presented in the obstetrics emergency department, due to experiencing escalating fatigue over the past three weeks and an apparent increase in skin paleness. Clinical assessments revealed her to be pale and feverish, with bruises on her forearms and body, but without signs of enlarged spleen or swollen lymph nodes. She presented a fever of 37.3 degrees Celsius, a heart rate (HR) of 118 beats per minute (bpm), a blood pressure of 126/72 mmHg and an oxygen saturation (SpO_2_) of 96%.

Blood tests indicated severe pancytopenia, with haemoglobin levels at 6.8 g/dL, a white blood cell count of 1,200/mm^3^ (with an absolute neutrophil count of 350/mm^3^) and platelets at 15,000/mm^3^, and in the blood smear, a mild macrocytosis was found, with a ferritin of 268 ng/mL (reference range: 10-150 ng/mL), vitamin B12 of 663 pmol/L (reference range: 180-668 pmol/L) and folic acid of 10 ng/mL (reference range: 4-17 ng/mL). The other laboratory test results were in the normal range with prothrombin time (PT) of 11.4 seconds, activated partial thromboplastin time (aPTT) of 32 seconds and fibrinogen of 500 mg/dL (reference range: 200-400 mg/dL).

No anomalies were detected in a pelvic ultrasound, and her medical and family histories were clear of any previously identified conditions. Blood work from earlier in the pregnancy was normal, and current tests showed standard levels of ferritin, vitamin B12 and folic acid, with liver and viral screenings returning no irregularities.

**Figure 1:**
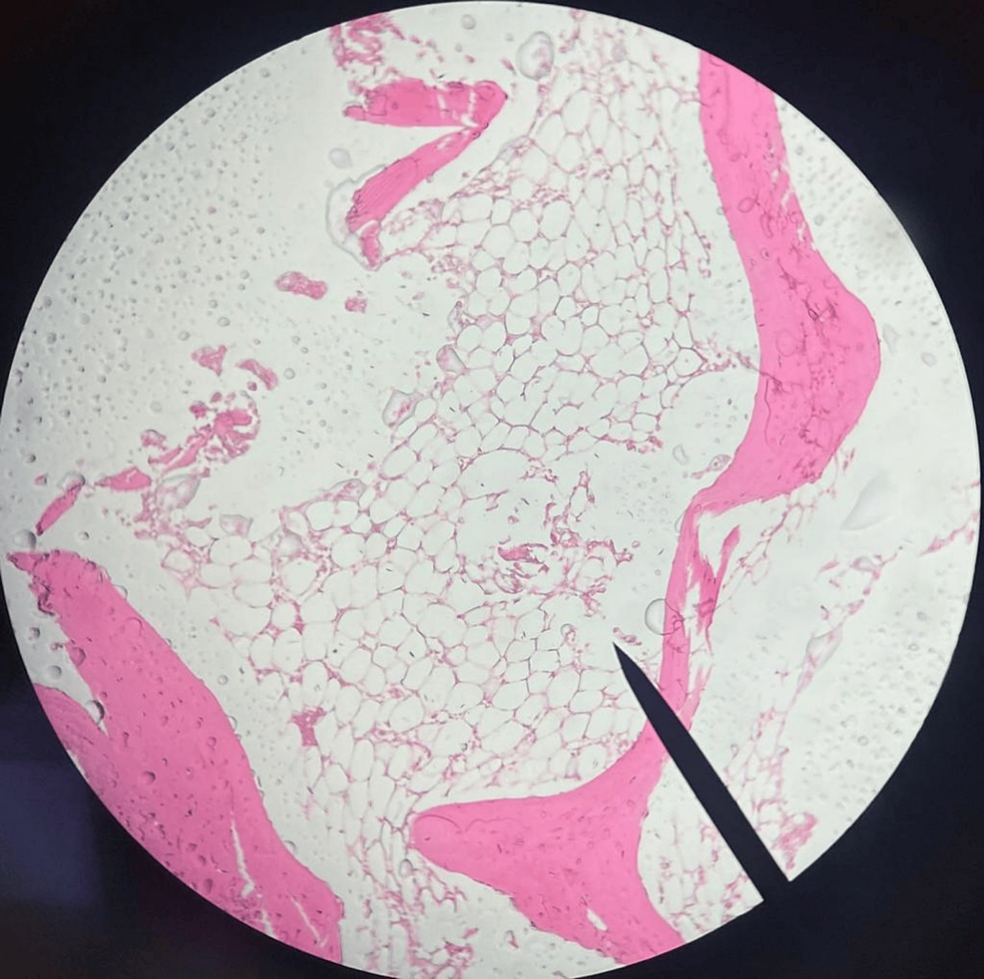
Bone marrow markedly hypocellular

Consultation with a haematologist led to a bone marrow aspiration and flow cytometry analysis, which highlighted a significant decrease in bone marrow cellularity without signs of abnormal cell growth or dysplasia. A further bone marrow biopsy confirmed a cellularity level of 15%, absent of fibrosis or abnormal cell proliferation (Figure 1). These clinical observations and diagnostic findings together led to the diagnosis of severe aplastic anaemia.

The patient was moved to the haematology department for specialised care. Given her pregnancy, selecting a treatment approach required careful consideration of both the urgent needs of the mother and the potential risks to the foetus. The initial treatment strategy included supportive measures such as regular transfusions of packed red blood cells and platelets, alongside fluconazole 100 mg daily, and also, Ceporin prophylaxis was used. Throughout the treatment, the patient's foetal development was meticulously monitored and maintained through platelet transfusions, a PLT of >20,000/mm^3^ to perform spontaneous delivery.

The baby was delivered at 39 weeks and four days, weighing 3,100 g. No abnormalities were found. Deviation from the norm in lochia production was not reported. The baby's blood test was performed, and all resulted in normal range. Despite a three-week postpartum observation period in the hospital, the patient's blood condition showed no signs of improvement.

Subsequently, the medical team recommended starting cyclosporine treatment, which the patient agreed to. Nonetheless, after being discharged, she decided against following the prescribed medication regimen. Regrettably, she later declined further medical assistance or hospitalisation and passed away at home after two months from the moment of giving birth to the baby.

## Discussion

The complex interplay between aplastic anaemia (AA) and pregnancy introduces significant challenges across both medical and obstetrical disciplines. This review delves into the current research concerning the causes, mechanisms, diagnosis, therapies and prognoses of AA within the context of pregnancy.

Causes of AA in pregnancy

The emergence of AA during pregnancy is influenced by a blend of factors, including immune responses, exposure to medications and viral infections, with particular attention to how pregnancy-related immune adjustments might trigger AA.

Immune Responses

Immune system modifications play a pivotal role in AA's development, often classified as an autoimmune condition [13]. Pregnancy introduces a period of heightened immune alteration, aimed at safeguarding the foetus whilst maintaining the mother's ability to counter infections. This state of immune flux may, in some instances, lead to autoimmune reactions, precipitating conditions such as AA [13].

Medication Exposure

The intake of certain medications during pregnancy is known to contribute to AA by causing bone marrow suppression. This includes nonsteroidal anti-inflammatory drugs, specific antibiotics, antithyroid medications and antiepileptic drugs [14]. Thus, vigilant management of a pregnant patient's drug regimen is crucial to decrease the risk of AA.

Viral Infections

Recognised as a causative factor for AA, infections by viruses such as Epstein-Barr virus, hepatitis viruses, HIV and parvovirus B19 can either directly harm hemopoietic stem cells or incite a maladaptive immune reaction [13,14]. Effective infection prevention and management are essential in averting AA during pregnancy.

These elements underscore a critical connection to the immunological alterations induced by pregnancy, which might either initiate or exacerbate AA's pathological processes in susceptible females [12,13]. Investigating these causative factors further is vital for enhancing our comprehension and treatment of AA in the pregnant population. A more nuanced understanding of these mechanisms could lead to targeted treatment strategies and improved prognostic outcomes, enriching the care and prognosis of AA during pregnancy.

Pathophysiology of AA in pregnancy

The pathophysiology of aplastic anaemia (AA) during pregnancy involves the bone marrow's failure to produce adequate blood cells, leading to pancytopenia, and a notable reduction in bone marrow cell activity, known as hypocellularity [1]. Unlike genetic disorders, AA is typically acquired, stemming from immune reactions, exposure to specific medications and certain viral infections [14]. The intersection of pregnancy with AA has intrigued researchers since Paul Ehrlich's first report of the condition in 1888, in which the patient, who was pregnant, succumbed to postpartum haemorrhage a month after giving birth [2].

The link between pregnancy and AA remains somewhat elusive despite the condition's recognition over a century ago. Initial investigations have not conclusively determined a correlation between pregnancy and increased AA risk [14].

However, contrasting evidence points towards a potential direct link between pregnancy and AA onset. Pregnancy is known for its profound hormonal and immune alterations. The hypothesis that these changes might aggravate AA's manifestations is subject to ongoing investigation, with the precise mechanisms yet to be definitively established [13,14].

Although the exact relationship between pregnancy and AA remains to be fully unraveled, the variance in research findings highlights the complexity of this interaction and underscores the necessity for continued exploration to thoroughly comprehend AA's pathophysiology in pregnant individuals.

Diagnosis of AA in pregnancy

Diagnosing aplastic anaemia (AA) in pregnant individuals often presents considerable challenges, with the standard diagnostic framework largely relying on laboratory tests that reveal pancytopenia, a decrease in red blood cells, white blood cells and platelets frequently associated with AA, and to confirm AA, a bone marrow biopsy is typically used [15]. Nevertheless, the procedure's invasiveness raises significant safety concerns for the expectant mother and her foetus. The risks associated with bone marrow biopsy, including infection, bleeding and pain, coupled with the potential for indirect foetal harm through maternal distress and infection risk, necessitate cautious consideration [15,16].

These risks underscore a dilemma in treating pregnant patients with AA: the imperative for precise diagnosis to guide treatment against the imperative to minimise foetal risk. Consequently, there is a broad medical consensus to defer bone marrow biopsies during pregnancy unless critically necessary [15].

The urgency for alternative diagnostic techniques that are safer and less invasive is clear, highlighting a pressing need for research into novel approaches suitable for pregnant patients [14]. Such advancements would aim to maintain diagnostic accuracy without compromising the well-being of both the mother and foetus, addressing a crucial need in medical practice and research for enhanced safety and efficacy in diagnosing AA during pregnancy.

Treatment of AA in pregnancy

Treating aplastic anaemia (AA) in pregnant females involves navigating the delicate balance between effective treatment and ensuring the safety of the foetus. Immunosuppressive treatments, a cornerstone of AA therapy, carry teratogenic risks that could impact foetal development negatively [17,18]. Similarly, bone marrow transplantation (BMT), whilst a potentially curative option, introduces significant risks to both the mother and the unborn child [19].

As a result, a more conservative management strategy, emphasising supportive care such as blood transfusions and growth factor administration, may be preferable. Although this approach does not tackle AA's root cause, it aims to alleviate symptoms and maintain the patient's health, posing fewer risks to the pregnancy. The success of this strategy can vary, influenced by the disease's severity and the patient's overall health status.

BMT offers a potential cure for AA by replacing malfunctioning marrow with healthy marrow from a donor. However, its application during pregnancy is fraught with challenges due to the intense conditioning treatments required, such as high-dose chemotherapy and/or radiation, which carry risks of miscarriage, birth defects, growth restriction in the foetus, premature birth and potential long-term developmental issues for the child, facing significant risks from the treatment, including infertility, infections, graft-versus-host disease and even mortality [19]. Decisions around BMT during pregnancy thus require careful consideration of various factors, including the mother's health, the severity of AA, treatment alternatives and the stage of pregnancy.

The literature on using BMT for AA acquired during pregnancy is sparse, highlighting the urgent need for more research to ascertain its safety and effectiveness and to inform treatment guidelines for this specific group [19].

Outcomes for pregnant females with AA and their babies depend greatly on the disease's severity and how quickly it is diagnosed. Early detection and appropriate management are crucial in improving these outcomes, emphasising the importance of prompt medical attention for pregnant females showing symptoms of AA.

This discussion underscores significant gaps in our understanding of AA during pregnancy, spanning its causation, progression, diagnosis, treatment and effects on pregnancy outcomes. Filling these gaps through further research is essential for enhancing clinical practices and outcomes for pregnant females diagnosed with AA.

A comprehensive clinical approach for a pregnant female with AA should engage a multidisciplinary team, including haematologists, obstetricians and neonatologists, focusing on the best possible outcomes for both the mother and her baby.

## Conclusions

In pregnant individuals, aplastic anaemia (AA), a critical and rare condition characterised by bone marrow failure and comprehensive blood cell deficiency, poses unique challenges within the medical and obstetric realms. This disorder's exacerbation during pregnancy is attributed to a combination of immune responses, exposure to certain medications and viral infections, further complicated by the physiological changes occurring during this period. The diagnosis process for AA in expectant mothers is intricate, initially relying on laboratory evidence of pancytopenia, a notable decrease in blood cells, and subsequently confirmed through a decrease in marrow cellularity via bone marrow biopsy. The management of AA in this population is fraught with difficulty, primarily due to the teratogenic potential of many immunosuppressive medications and the risks associated with bone marrow transplantation, making supportive care a critical component of treatment, albeit its success varies with the patient's health status and the severity of the condition. The prognosis for both the mother and the foetus greatly depends on how swiftly and accurately AA is diagnosed and managed, emphasising the importance of immediate and attentive care for pregnant patients suspected of having AA. Although current studies provide some understanding of AA in pregnancy, they also point to significant gaps in knowledge, necessitating further research to enhance patient care. For the most effective management and outcomes, a collaborative approach involving specialists in haematology, obstetrics and neonatology is advocated.
